# Editorial: Peptides against infectious diseases: from antimicrobial peptides to vaccines

**DOI:** 10.3389/fphar.2024.1522148

**Published:** 2024-12-11

**Authors:** Joanna Bojarska, Xiumin Wang, Mariusz Skwarczynski

**Affiliations:** ^1^ Department of Chemistry, Technical University of Lodz, Lodz, Poland; ^2^ Institute of Feed Research, Chinese Academy of Agricultural Sciences, Beijing, China; ^3^ Key Laboratory of Feed Biotechnology, Ministry of Agriculture and Rural Affairs, Beijing, China; ^4^ School of Chemistry and Molecular Biosciences, The University of Queensland, St Lucia, QLD, Australia

**Keywords:** peptides, antimicrobial peptides, infectious diseases, vaccines, short peptides

A remarkable progress in the development of peptide-based therapeutic agents has been observed. Notably, they represent ∼10% of the total drug market. Peptides, typically defined as sequences consisting of two to fifty amino acids, are stand-alone entities or parts of proteins that are responsible for a vast variety of bio-functions. In other words, peptides link features of small chemical molecules and biologics; hence, their functions are difficult to imitate by traditional molecules. Peptides, both short and long peptides, have unique features. They are fully biodegradable, biocompatible, highly selective in biological interactions, and normally safe for living organisms. They are the most versatile molecules with the greatest structural and functional diversity. We can say that peptides are “smart by nature” (Apostolopoulos et al., 2021; Bojarska et al., 2021; D'Aloisio et al., 2021; Al Musaimi et al., 2022). Notably, peptides derived from sources other than proteins also serve as vital bio-agents, including hormones, components of innate immunological systems (antimicrobial peptides), neuropeptides, cell-penetrating peptides, and enzyme inhibitors. Thus, not surprisingly, peptides and peptidomimetics with diverse physiological functions have been widely utilized as therapeutic agents.

Among other applications, they have been employed to detect, prevent, and treat infectious diseases. Peptides are used in diagnostic applications, imitating the molecular recognition properties of proteins for the detection of bacterial or viral infections, as antibiotics mimicking the function of natural antimicrobial peptides, and as crucial vaccine antigenic components and immunostimulators, to name a few applications. They are a very promising platform for drug and vaccine development (Skwarczynski and Toth, 2016; Wang and Li, 2023). Nevertheless, peptides do have certain limitations–they are easily biodegradable, break all Lipinski’s rules, lack oral activity, and peptide antigens exhibit poor immunogenicity (Apostolopoulos et al., 2021; Apostolopoulos et al., 2022; Bojarska et al., 2021; Bucci et al., 2021). Thus, various strategies have been developed to overcome these shortcomings by structural modification or utilization of specialized formulations.

This Research Topic, aimed to provide a forum for new studies and discussions on novel concepts, mechanisms, and applications in the field of peptide-based detection, prevention, and treatment of infectious diseases. It includes five original articles, one brief research report, and one review paper.

Li et al. conducted a comprehensive review of the current research progress in the development and application of polyethylene glycol (PEG)-based therapeutic proteins and peptides. The authors discussed either the physicochemical properties or classification of PEG derivatives, keeping in mind the prospects of PEGylated peptides. In addition, they show opportunities for further innovations in this area.

Cationic ultrashort lipopeptides are promising agents to fight multidrug-resistant bacteria. Hernández-Ortiz et al. demonstrated that anionic and neutral zwitterionic tripeptides exhibit antimicrobial and membrane-disrupting effects against *inter alia Streptococcus pneumoniae* and *Streptococcus pyogenes*. For their experiment, the researchers utilized newly designed C-terminal lipidated tripeptides with specific features (net charges ranging from −2 to +1) and a fatty amine conjugated to the C-terminal. Thus, the authors presented lipopeptides as appealing antimicrobial candidates with enhanced bioactivity and reduced toxicity for potential use in medical applications.

*Candida* species are fungus species that have the ability to form biofilms that contribute to their pathogenicity and provide protection against antifungal drugs. Among these species, *Candida glabrata* is a prevalent yeast responsible for infections in humans, and is known for its ability to form biofilms. Madhuri et al. evaluated the combined effects of two highly promising naturally occurring antifungal lipopeptides named AF4 and AF5. They reported that these peptides exhibited an enhanced *in vitro* anti-biofilm effect when used alone or in combination with fluconazole.

Melittin, an antimicrobial peptide derived from bee venom, is generally considered toxic and consequently lacks clinical utility. Nevertheless, modifications on its amino acid sequence through cyclization, dimerization, or truncation have been shown to reduce its toxicity. Matthyssen et al. substituted the three lysine residues with lysine homologs containing shorter side chains (such as ornithine, diaminobutyric acid, and diaminopropanoic acid) and dimerized melittin. They observed improved antimicrobial activity against Gram-negative bacteria, but no significant improvement in activity against Gram-positive bacteria. On the other hand, toxicity toward HEK293 and H4IIE cells was only slightly reduced. Furthermore, researchers suggested that more drastic modifications as well as encapsulation in nanocarriers, conjugation to other substances, and synergistic effects of melittin with antibiotics have the potential to reduce the effective dose of melittin to non-toxic levels.

*Acinetobacter baumann*i is a highly drug-resistant pathogen, posing significant challenges in clinical treatment. Meng et al. conducted an evaluation of the synergistic antibacterial activity and additive effects of peptide antibiotics in combination with other antimicrobial agents against A. *baumann*i in both planktonic and biofilm forms *in vitro*. More specifically, the combination of polymyxins B and E together with imipenem act synergistically, while polymyxin E with rifampicin and bacitracin with imipenem or meropenem demonstrated additive effects against bacteria in planctonic form. In antibiofilm activity, synergistic activity was observed for combination of polymyxin B and E with azithromycin, while additive effect was confirmed for mixture of teicoplanin with tigecycline or rifampicin. The use of polymyxins along with carbapenems or azithromycin was suggested to represent an optimal therapeutic approach for *A. baumannii* infections.

Suppressive antibiotic therapy is a valuable strategy to reduce infection progression in situations where alternative therapeutic approaches are not viable. The 15 years old lipoglycopeptide antibiotic, dalbavancin, exhibits a prolonged half-life and allows for less frequent dosing. Ruiz-Sancho et al. present their multicenter real-life clinical experience across multicenters highlighting the efficacy of dalbavancin-based approach in suppressive antibiotic therapy for prosthetic joint or vascular infections. Their findings provide valuable insights to support further evaluation of this treatment modality in patients with prosthetic infections in the outpatient setting when other methods are not feasible.

Thanatin, a cyclic analog of a beta-hairpin antimicrobial peptide, has diverse antibacterial and antifungal activities. It forms transmembrane pores and inhibits outer membrane biogenesis by binding to the LptA protein of *Escherichia coli* and blocking lipopolysaccharide transport. Notably, thanatin acts in synergy with antibiotic polymyxin B. Shepperson et al. developed thanatin analogs in which labile disulfide was replaced with vinyl sulfide bridge mimetics. This substitution strategy is promising for the development of narrow-spectrum antimicrobial agents that exhibit limited propensity for resistance emergence in medical environments.

To conclude, the studies featured in this Research Topic have significantly enhanced the potential of peptides for therapeutic applications in infectious diseases through structural and other modifications. The studies presented in this Research Topic have highlighted directions for enhancing the therapeutic potential of peptides in treating infectious diseases, through the discovery of novel peptides, the introduction of structural modifications in peptides, and by applying peptides in combination therapies.
